# Improved prediction of 5-year mortality by updating the chronic related score for risk profiling in the general population: lessons from the italian region of Lombardy

**DOI:** 10.3389/fpubh.2023.1173957

**Published:** 2023-08-30

**Authors:** Giovanni Corrao, Andrea Stella Bonaugurio, Yu Xi Chen, Matteo Franchi, Antonio Lora, Olivia Leoni, Giovanni Pavesi, Guido Bertolaso

**Affiliations:** ^1^National Centre for Healthcare Research and Pharmacoepidemiology, University of Milano-Bicocca, Milan, Italy; ^2^Unit of Biostatistics, Epidemiology and Public Health, Department of Statistics and Quantitative Methods, University of Milano-Bicocca, Milan, Italy; ^3^Lombardy Region DG Welfare, Milan, Italy

**Keywords:** score, stratification, comorbidities, healthcare, risk profiling

## Abstract

**Objective:**

The aim of this study was to improve the performance of the Chronic Related Score (CReSc) in predicting mortality and healthcare needs in the general population.

**Methods:**

A population-based study was conducted, including all beneficiaries of the Regional Health Service of Lombardy, Italy, aged 18 years or older in January 2015. Each individual was classified as exposed or unexposed to 69 candidate predictors measured before baseline, updated to include four mental health disorders. Conditions independently associated with 5-year mortality were selected using the Cox regression model on a random sample including 5.4 million citizens. The predictive performance of the obtained CReSc-2.0 was assessed on the remaining 2.7 million citizens through discrimination and calibration.

**Results:**

A total of 35 conditions significantly contributed to the CReSc-2.0, among which Alzheimer's and Parkinson's diseases, dementia, heart failure, active neoplasm, and kidney dialysis contributed the most to the score. Approximately 36% of citizens suffered from at least one condition. CReSc-2.0 discrimination performance was remarkable, with an area under the receiver operating characteristic curve of 0.83. Trends toward increasing short-term (1-year) and long-term (5-year) rates of mortality, hospital admission, hospital stay, and healthcare costs were observed as CReSc-2.0 increased.

**Conclusion:**

CReSC-2.0 represents an improved tool for stratifying populations according to healthcare needs.

## 1. Introduction

There is a need for profiling, mapping, and checking the health needs of the general population (i.e., citizens who receive assistance from a given health system, each of whom is denoted as a system beneficiary) over time to identify who and count how many among the system beneficiaries need to receive healthcare, sometimes intensively, sometimes early, depending on their clinical complexity, and the outcome of interest. Several risk scores have been developed for this purpose. A recent systematic review identified 35 currently available scores, and most of them were used for predicting mortality as well as for the prediction of healthcare use or healthcare costs, hospital admission, or health-related quality of life. Moreover, most scores were based on pathologies/conditions (weighted or in combination with other parameters), while others used diagnostic categories, drug use, or physiological measures (1). Among these, the Chronic Related Score (CReSc) proposed the idea of profiling universal health system beneficiaries by investigating past use of healthcare, diagnoses, and medications to identify predictors of adverse events, such as mortality, hospital admissions, and high costs (2). Although CReSc is a tool that is able to finely predict mortality, costs, and hospital admissions, its original form has at least two substantial limitations. First, the score was based on algorithms for tracing 65 diseases/conditions and was developed more than 10 years ago; however, new drugs and diagnostic-therapeutic strategies have since been developed, and an update to more modern tracing algorithms is therefore appropriate. Second, although the coexistence of both physical and mental illness within multimorbidity is established to be prevalent (3), to the extent that the need to account for mental disorders in multimorbidity studies was highlighted in a recent report (4), the original form of CReSc did not include mental disorders among the 65 diseases/conditions used in its development.

Given these premises, a working group was appointed by the Regional Health Authority of Lombardy to develop a new version of CReSc by updating the algorithms for tracing the diseases/conditions composing the score, including mental disorders. Here, we describe the tracing algorithms, validation tools, and predictive properties of the new version of CReSc, denoted CReSc-2.0.

## 2. Materials and methods

### 2.1. Target population and data sources

Citizens who, on 1 January 2015, (i) were residents in Lombardy (*n* = 9,990,817), (ii) had been beneficiaries of the Regional Health Service for at least 5 years (*n* = 9,793,138), (iii) were aged 18 years or older (*n* = 8,186,373), and (iv) were not institutionalized (*n* = 8,126,693) formed the target population of 8.1 million citizens (approximately 14% of the Italian population of that age group) (Supplementary Figure 1). In Italy, all citizens have equal access to healthcare provided by the National Health Service (NHS). An automated system of Healthcare Utilization (HCU) databases is used to manage health services in each region, including Lombardy. HCU data include a variety of information on NHS beneficiaries, such as diagnosis at discharge from public or private hospitals, outpatient drug prescriptions, specialist visits, and diagnostic exams provided fully, or in part, free-of-charge by the NHS, and ascertained chronic illness benefit payment exemption codes. In addition, a specific information system concerning mental healthcare gathers data from regional Departments of Mental Health accredited by the NHS. These various types of data can be interconnected since a unique individual identification code is used by all databases for each NHS beneficiary.

### 2.2. Privacy-by-design issues

Privacy-by-design (or data-protection-by-design) principles were adopted in conducting the current study (5–7). In general, safe settings and computing were both ensured by regional infrastructure, which provided a safe environment to store health data and prevented unauthorized imports and exports of individual records. Safe data were ensured by the pseudo-anonymization of identification codes, with the inverse process being allowed only by the Regional Health Authority on request from judicial authorities. Furthermore, to prevent individual identification, outputs were exported, provided that more than three individual records typified each specific category by which beneficiaries were stratified. The safety of the people was guaranteed by the assurance that only a few researchers (AB and YC, with the supervision of MF), who complied with ethical data usage and legal agreements, were authorized to conduct data analysis, that is, to submit SAS macrocodes to the database previously prepared by the regional staff and export the outputs, without any possibility of accessing/seeing the individual records directly. Finally, the safety of the project was ensured by the Regional Health Authority of Lombardy's assessment of the potential public benefits. Secondary data, such as ours (i.e., healthcare utilization data that was primarily collected for repaying healthcare providers), should only be used for secondary purposes provided that the data owner (in our case, the regional authority) realizes that the purpose is useful for generating knowledge aimed at improving the quality of care, ensuring a safe project should be a priority in the field of privacy-by-design. A comprehensive overview of “privacy research environments by design,” which is the most common approach used in the United Kingdom and mainland Europe, as well as inspiring the use and interconnection of secondary data, is reported elsewhere (8).

### 2.3. Updating the score

Chronic conditions, which formed the starting point for the development of the original CReSc (2), were revised by two members of our team (OL and AL), in order to update the algorithms to capture patients with (i) the 65 chronic conditions on the original list through the identification of healthcare strategies introduced in the last 10 years, including approved drugs and new diagnostic-therapeutic pathways recommended by qualified guidelines; (ii) four severe mental disorders that were omitted from the original list, including major depression, schizophrenia, bipolar disorder, and personality disorder. The new algorithms for capturing each of the 69 chronic conditions in CReSc-2.0 are reported in the Supplementary material.

To select conditions that could independently predict 5-year mortality (i.e., the main outcome of interest), the study proceeded as follows. First, two out of three of the 8.1 million citizens forming the target population (i.e., almost 5.4 million citizens) were randomly selected to form the training set. These patients were followed until the earliest date between death and censoring (emigration or 31 December 2019). Second, a Cox proportional hazard regression model was fitted to compute hazard ratio (HR) values, estimating the relationship between the selected covariates and the time of death (9). Covariates included in the model were sex, age (on 1 January 2015), and the 69 candidate predictors, where the latter were entered in the model as dichotomous variables with values of 1 or 0, according to whether the specific condition was or was not recorded at least once in the 10 years prior to baseline. Third, the least absolute shrinkage and selection operator method was applied to select conditions able to independently predict 5-year mortality (10). Finally, a weight was assigned to each of the selected conditions by multiplying the coefficients estimated from the model by 10 and rounding the product to the nearest whole number (11). The weights thus obtained were then summed to produce a total aggregate score. To simplify the system in order to account for excessive heterogeneity of the total aggregate score, the latter was categorized by assigning increasing values of 0, 1, 2, 3, and 4 to the aggregate score categories of 0, 1–10, 11–20, 21–30, and ≥31, respectively. The index obtained in this way was denoted the “Updated Chronic Related Score” (CReSc-2.0). The five levels of CReSc-2.0 denoted citizens who required increasing clinical attention and healthcare resources and were categorized as citizens who have (i) no evidence of chronic comorbidity, who thus require interventions of primary prevention and health promotion (CReSc-2.0 = 0); (ii) at least one “not severe” chronic comorbidity, thus requiring health lifestyle recommendations and clinical monitoring (CReSc-2.0 = 1); (iii) at least one “more severe” chronic comorbidity, thus requiring NHS oversight and personalized healthcare programs (CReSc-2.0 = 2); (iv) comorbidity related to higher clinical complexity, thus requiring careful clinical monitoring (CReSc-2.0 = 3); and (v) more severe clinical conditions urgently requiring intensive care (CReSc-2.0 = 4). A pyramidal representation, cross-sections of which show the proportion of NHS beneficiaries belonging to each CReSc-2.0 level, was built to inform stakeholders of healthcare needs.

### 2.4. Exploring score performance

Weights obtained from the training set were applied to a validation set consisting of NHS beneficiaries who were not included in the training set (i.e., 2.7 million individuals).

Two metrics were used to evaluate the predictive performance of the score. First, discrimination, indicating how well the model can distinguish individuals with an outcome from those without the outcome, was measured using receiver operating characteristic (ROC) curves and the corresponding area under the ROC curve (AUC) values (12). Second, calibration, ascertaining the concordance between the model's predictions and observed outcomes, was evaluated using a calibration plot. The plot displayed predicted vs. observed 5-year survival probabilities for individuals with increasing predicted risk. Differences in predicted and observed frequencies were assessed in the total cohort, indicating the extent to which predictions were systematically too high or too low (referred to as calibration-in-the-large) and the recalibration slope, reflecting the slope of the calibration plot and ideally equal to one (13).

### 2.5. Secondary outcomes

Secondary analyses were performed to verify the robustness of CReSc-2.0 in predicting outcomes other than cumulative 5-year all-cause mortality. With this aim, cumulative healthcare costs, rates of hospital admissions, and cumulative days of hospital stays were calculated along the increasing CReSc categories for the entire 5-year time window. Finally, the rates of hospital admission and cumulative days of hospital stays, both expressed as an average number every 1,000 person-years (PY), were considered.

## 3. Results

### 3.1. CReSc-2.0

The 35 conditions that significantly and independently contributed to the CReSc-2.0 and the corresponding burdens, that is, the global health implications obtained from their weights (i.e., the strength of the associations with 5-year mortality), prevalence (i.e., the number of citizens affected), and costs (i.e., NHS expenditure on caring for patients with each condition), are reported in Table 1. Almost 36% of NHS beneficiaries had at least one condition contributing to the CReSc-2.0. Alzheimer's disease, dementia, and heart failure contributed most to the total aggregate score. As expected, with respect to the other listed conditions, hypertension, depressive disorders, and type 2 diabetes had higher prevalence rates, while dialytic treatment, HIV/AIDS, acromegaly, and gigantism had higher per-capita healthcare costs. Considering prognosis, prevalence, and costs as a unique proxy of disease burden, heart failure, active neoplasia, arrhythmic myocardiopathy, ischaemic cardiopathy, and chronic obstructive pulmonary disease showed the highest impacts in our setting.

**Table 1:** Weights, prevalence, and burden of conditions contributing to the updated Chronic Related Score (CReSc-2.0).

	**Disease/condition^a^**	**CReSc-2.0 Weight^b^**	**Prevalence rate (%)**	**Per-capita annual cost (€)**	**Burden ranking^c^**
1	Alzheimer's disease	22	0.26	9,712	32
2	Dementia	20	0.22	11,905	22
3	Heart failure	19	2.06	21,802	1
4	Neoplasia, active	15	1.94	23,011	2
5	Parkinson's disease	12	0.40	18,841	11
6	Dialysis	12	0.09	140,968	12
7	Arrhythmic myocardiopathy	11	2.59	15,443	3
8	Hypertension	10	15.07	9,833	18
9	Cerebral vasculopathy	8	1.80	16,619	7
10	Chronic obstructive pulmonary	8	1.94	18,894	5
11	Ischemic cardiopathy	8	2.91	16,198	4
12	Neoplasia, follow-up	8	2.87	14,805	8
13	Respiratory insufficiency/oxygen therapy	8	0.12	23,942	13
14	Liver cirrhosis	8	0.40	28,513	8
15	Neoplasia, remission	7	2.31	12,348	14
16	Chronic kidney failure	6	0.96	24,791	6
17	No-arrhythmic myocardiopathy	6	2.91	14,826	10
18	Diseases of the nervous system and sense organs	5	0.13	14,785	21
19	Myasthenia gravis	5	0.03	16,985	26
20	Depressive disorder	5	5.96	11,038	20
21	Diseases of the skin and subcutaneous tissue	5	0.02	26,446	30
22	Systemic sclerosis	4	0.06	12,438	34
23	Type II diabetes mellitus, complicated	4	0.54	28,987	15
24	Type II diabetes mellitus	4	5,75	14,564	17
25	Acromegaly and gigantism	4	0.02	54,094	35
26	Schizophrenic disorder	5	0.50	12,812	27
27	Epilepsy	4	0.65	13,102	25
28	Bipolar disorder	4	0.47	11,960	29
29	Arterial vasculopathy	4	0.98	23,041	16
30	Chronic pancreatitis	4	0.05	16,010	31
31	Venous vasculopathy	3	0.39	19,349	24
32	Rheumatoid arthritis	3	0.45	28,592	19
33	HIV-positive and full-blown AIDS	2	0.34	53,637	23
34	Valvular cardiopathy	1	0.70	25,012	28
35	Chronic hepatitis	1	1.02	14,513	33
	Beneficiaries with at least one selected disease/condition	-	36.13	-	-

a Disease/condition selected as an independent predictor of 5-year mortality.

b Weight is calculated by multiplying the specific coefficient of the survival model by 10 and rounding it to the nearest whole number.

c Ranking obtained by multiplying the CReSc-2.0 weight ranking, prevalence rate ranking, and per-capita annual cost ranking of each selected disease/condition.

**Figure 1:**
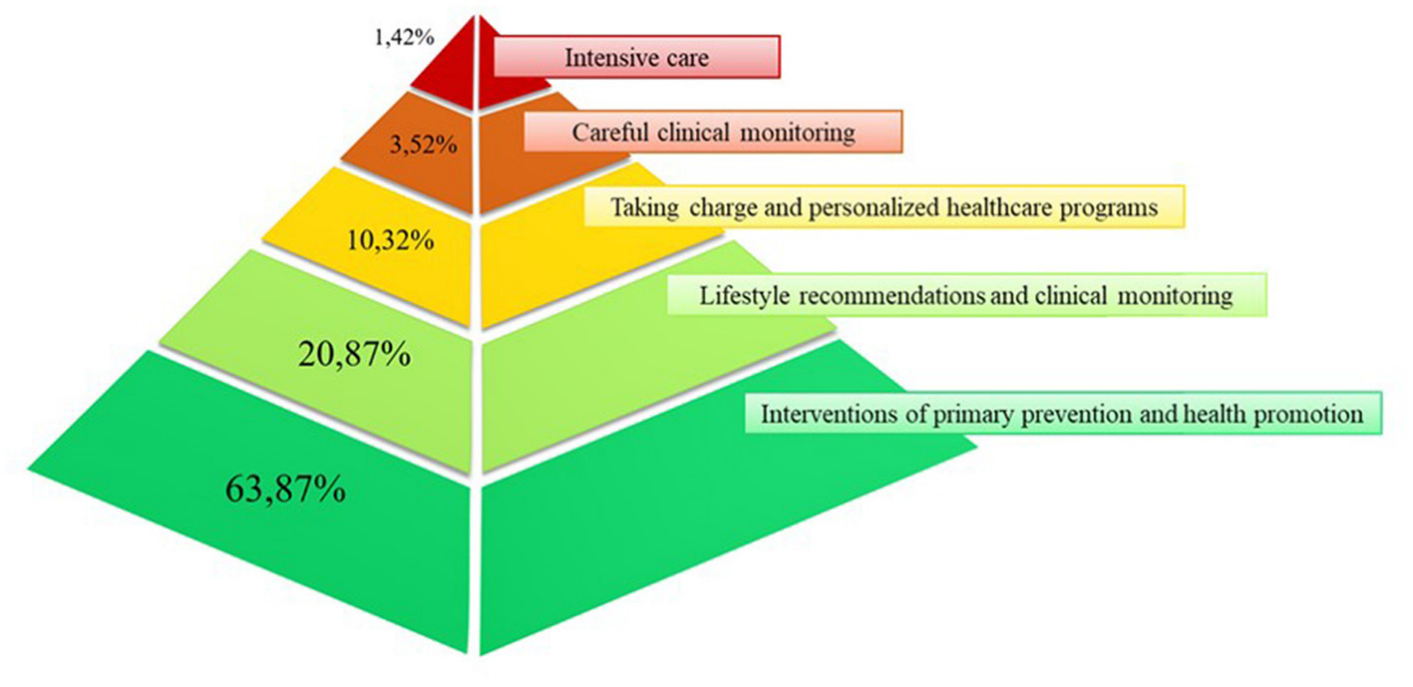
Pyramid illustrating the distribution of National Health Service beneficiaries in Lombardy, Italy, according to the updated Chronic-Related Score (CReSc-2.0).

The pyramidal structure of the CReSc-2.0 distribution is illustrated in Figure 1. The data indicate that primary prevention and health promotion interventions (CReSc-2.0 = 0) should be addressed to less than two-thirds (64%) of citizens. Lifestyle recommendations and clinical monitoring (CReSc-2.0 = 1) (1), oversight and personalized healthcare programs (2), careful clinical monitoring (3), and intensive care (4) were required by 1 in 5, 1 in 10, 1 in 30, and 1 in 67 citizens, respectively.

**Figure 2:**
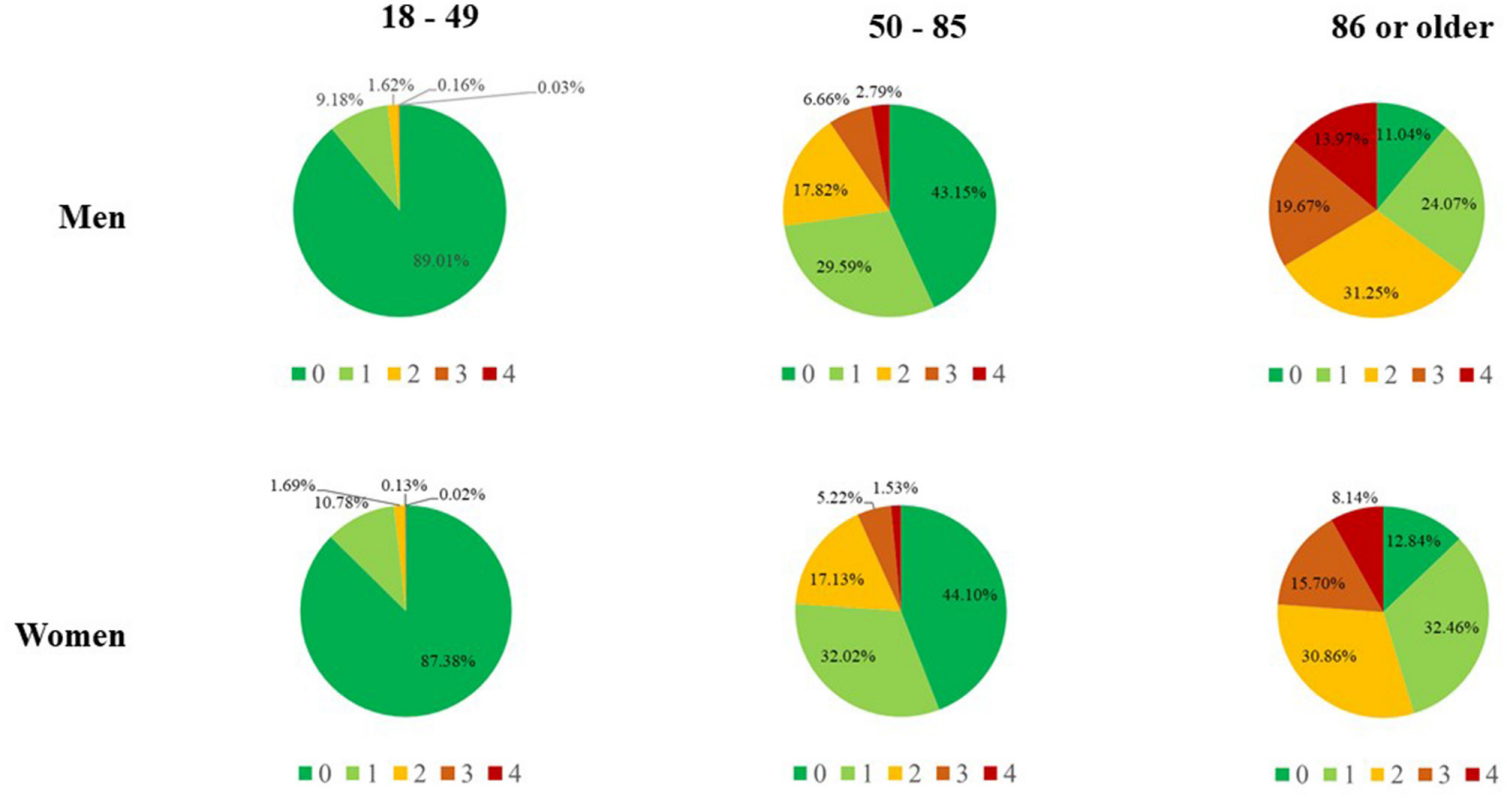
Updated Chronic Related Score (CReSc-2.0) among National Health Service beneficiaries of Lombardy, Italy, according to sex and age categories.

CReSc**-**2.0 clearly demonstrated that men and older adults had worse clinical status than women and younger people (Figure 2), as the prevalence of NHS beneficiaries with at least one chronic disease contributing to the score increased progressively with age in both women (from 12.6 to 87.2%) and even more in men (from 11.0 to 89.0%).

### 3.2. CReSc-2.0 performance

**Figure 3:**
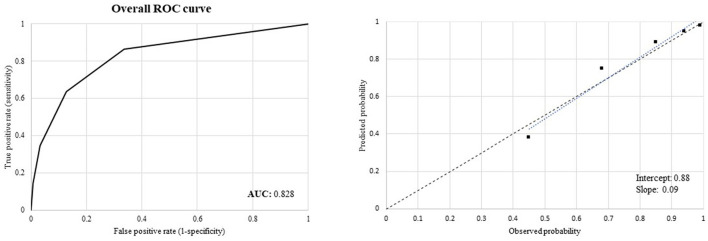
Receiver Operating Characteristics (ROC) curves to assess discriminant power **(left box)** and calibration plots comparing observed and predicted 5-year survival probabilities of the updated Chronic Related Score (CReSc-2.0) **(right box)**.

Our score had very good predictive performance as suggested by (i) the discrimination power measured by ROC curve analysis (Figure 3 left box) and the corresponding AUC value of 0.828 and (ii) the good agreement between the observed and predicted survival probabilities, with values for the calibration-in-the-large analysis close to the ideal of zero (0.09) and values of the recalibration slopes close to the ideal value of one (0.88) (Figure 3 right box). The comparison of the discriminatory power between the CReSc-2.0 and the original CReSc is reported in Supplementary Table 2.

### 3.3. CReSc-2.0 predictability

**Figure 4:**
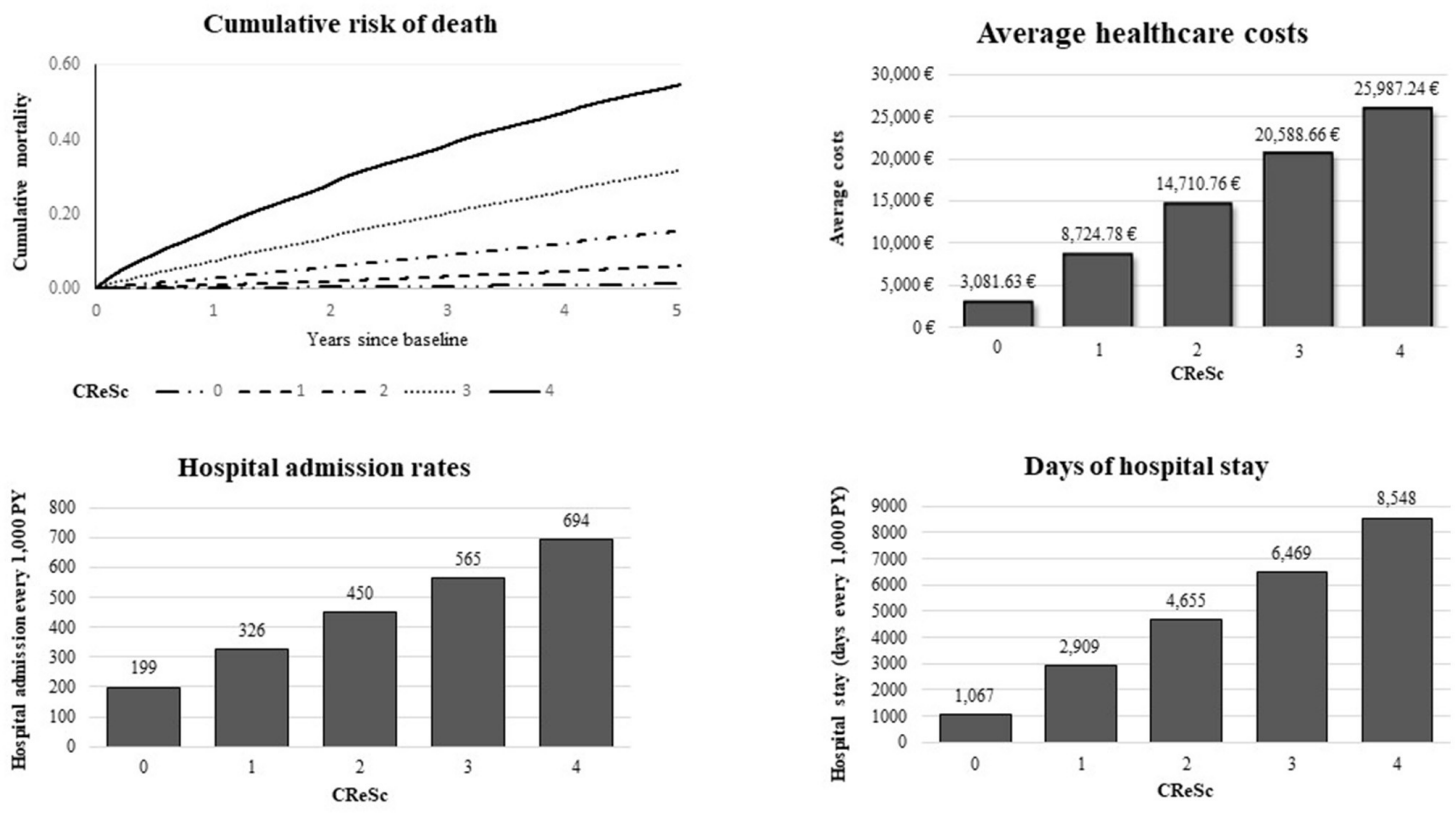
Five-year cumulative mortality and healthcare costs, rates of hospital admission, and days of hospital stay, according to the updated Chronic Related Score (CReSc-2.0) distribution.

A clear positive trend toward increasing rates of all the considered outcomes was observed as CReSc-2.0 increased (Figure 4). In particular, with respect to NHS beneficiaries with the lowest score (CReSc-2.0 = 0), those with the highest score (CReSc-2.0 = 4) had a higher 1-year risk of death, 5-year risk of death, 5-year healthcare costs, rate of hospital admissions, and rate of cumulative hospital stay at 107-fold (0.15 vs. 16%), 46-fold (1.2 vs. 55%), 8.4-fold (3,082 € vs. 25,987 €), 3.5-fold (199 vs. 694 hospital admissions every 1,000 PY), and 8.0-fold (1,067 vs. 8,548 days of stay every 1,000 PY), respectively.

## 4. Discussion

Our study shows that a score based on healthcare utilization data currently used for managing a regional universalistic health system such as Lombardy can stratify NHS beneficiaries according to their short-term (1-year) and long-term (5-year) outcomes, such as mortality, healthcare costs, and hospital admissions. As mortality is of most relevance to clinicians, whereas healthcare use and costs are more useful to healthcare providers, the versatility of our approach should be ensured by the ability of our score in predicting different outcomes. Future studies should investigate other outcomes relevant to patients, such as quality of life and self-reported health.

Our updated score significantly improved discriminatory power as measured by AUC values from 0.79 (CReSc) to 0.83 (CReSc-2.0). This improved score performance was facilitated by the updating of the algorithms used to identify NHS beneficiaries with chronic conditions, as well as by the inclusion of mental health disorders as candidate predictors of clinical and economic outcomes.

Detailed descriptions of all 69 chronic conditions considered in this study are provided in the Supplementary material to allow interested readers and stakeholders to replicate our findings and possibly suggest alternative, validated algorithms to trace chronic conditions. Meanwhile, to the best of our knowledge, no other tool with such high performance for measuring the health needs of the general population has been reported. Indeed, as previously reported (2), the original version of CReSc was shown to have a considerably higher discriminatory power as compared to the commonly used Charlson Comorbidity Index (14), indicating that the new CReSc-2.0 further increases the performance improvement as compared to other common indexes.

Our study also provides the following additional results. Among the four investigated mental illnesses, depressive, schizophrenic, and bipolar disorders contributed significantly and independently to the score, suggesting that mental health must be regarded as a major factor affecting health needs (15). The failure to select personality disorder is likely explained by its complex diagnostic classification and our consequent poor ability to identify relevant patients, rather than its poor prognostic weight (16). Notably, although depression strongly affected 5-year mortality (i.e., had a weight of 5, which was higher than that for physical illnesses, such as multiple sclerosis, rheumatoid arthritis, and chronic pancreatitis), and had a higher prevalence than other selected conditions (except for hypertension and type 2 diabetes), the costs of caring for patients with severe depression from the NHS perspective were lower than those for caring for patients with the other selected conditions (except for Alzheimer's disease and hypertension). Hence, the burden of severe depression (and other mental disorders) is lower than that of other conditions, but this is likely biased by the barriers and stigmatization experienced by patients with mental health disorders (17).

The present study has several strengths. First, because it was conducted in Italy, which has a publicly funded healthcare system involving virtually all citizens, our sample included all NHS beneficiaries, resulting in an unselected population and a very large sample size. Second, CReSc-2.0 was validated and tested on a random sample of almost 3 million NHS beneficiaries, a sample so large that random uncertainty only slightly affected our estimates. Finally, as the selected diseases were detected based on health services data, our findings overcome the false positive effects of socioeconomic inequalities in estimating chronic disease prevalence based on self-reports (18).

The potential limitations of our study must be considered when interpreting our findings. First, our scoring system did not capture health services supplied by private providers; this necessarily generates diagnostic misclassification. For example, patients with higher clinical complexity, mainly those requiring hospital admission, were more likely to be captured by our study. Yet, out-of-pocket healthcare payments were common during the study period, including in the context of universal coverage systems such as that in Italy (19), particularly for certain clinical areas, for example, psychiatric conditions (20). Furthermore, misdiagnosis (often due to poor accuracy in reporting diagnoses and comorbidities (21) and upcoding [sometimes in pursuit of higher reimbursements (22)] in hospital records may have generated overly conservative estimates of CReSc-2.0 performance; however, as diagnostic errors will have also influenced the compared diagnosis-based comorbidity scores, this issue does not undermine our main finding that CreSc-2.0 performs better than the original score, as well as other common comorbidity scores (14, 23, 24). Second, since the selected diseases/conditions (i.e., the outcomes contributing to CReSc-2.0) can be considered proxies of the quality of care (25), our scoring system may not be generalizable to other settings outside of Italy. Third, the split approach used in our study may also limit generalizability since randomly splitting the whole dataset into a training and a validation set has raised concerns from some authors (26). External validation in other settings (e.g., other Italian regions, countries outside Italy, and different calendar times) should be carried out to ensure the external validity of CReSc-2.0. Finally, we must be aware that CReSc-2.0 may not apply to every relevant outcome and cannot truly predict the individual conditions that increase the relative risk of death for a patient. For example, our score cannot take into account: (i) conditions that do not, or only marginally, affect 5-year mortality (e.g., type 1 and type 2 diabetes); (ii) NHS beneficiaries with a given condition who did not leave “footprints” of routine medical care able to detect that condition (e.g., untreated hypertension); and (iii) patients who did not survive for at least 2 years after the onset of an acute condition (e.g., fatal myocardial infarction).

In summary, we updated a prognostic score derived from data usually used for health system management in Lombardy, which is useful for predicting the short- and long-term mortality, hospitalization, and healthcare costs of each NHS beneficiary. CReSc-2.0 represents a useful tool for (i) policymakers, who need to address health policy and assess health system performance, by differentiating the intervention strategies in a coherent way with the health need and the consumption of the necessary resources, as well as for guaranteeing equity of access and homogeneity of taking care of patients based on their level of risk; (ii) clinicians, who need to detect and manage patients with frailty in everyday medical practice; and (iii) epidemiologists, who require an instrument for risk adjustment, based on clinical complexity, in clinical and epidemiological studies. We expect that the introduction of the CReSc-2.0 risk stratification tool may generate clinically and operationally important effects.

## Data availability statement

The data analyzed in this study is subject to the following licenses/restrictions: the data that support the findings of this study are available from Lombardy Region, but restrictions apply to the availability of these data, which were used under license for the current study, and so are not publicly available. Data are however available from the Lombardy Region upon reasonable request. Requests to access these datasets should be directed to DG Welfare Lombardy Region, https://www.regione.lombardia.it/wps/portal/istituzionale/.

## Ethics statement

Ethical approval was not required for the study involving humans in accordance with the local legislation and institutional requirements. Written informed consent to participate in this study was not required from the participants or the participants' legal guardians/next of kin in accordance with the national legislation and the institutional requirements.

## Author contributions

GC, AL, OL, GP, and GB contributed to conception and design of the study. AL and OL revised and updated the algorithms for defining the diseases and conditions for the development of the score. AB and YC performed the statistical analysis. MF supervised the analyses. GC wrote the first draft of the manuscript. All authors contributed to manuscript revision, read, and approved the submitted version.
