# Supplementary material for: Improved prediction of 5-year mortality by updating the chronic related score for risk profiling in the general population: lessons from the italian region of Lombardy
Source: Front Public Health. 2023 Aug 30;11:1173957. doi: 10.3389/fpubh.2023.1173957 (PMC10498767; doi:10.3389/fpubh.2023.1173957)
Supplement: Supplementary file 1 [file Data_Sheet_1.docx]

**Table S1. List of the 69 diseases and conditions candidate to be tested as predictors of five-year mortality.**

|  | **Disease/condition** |
| --- | --- |
| 1 | Active transplant recipients |
| 2 | Dialysis |
| 3 | Symptoms, signs and morbid conditions unspecified |
| 4 | Acromegaly and gigantism |
| 5 | Diseases of the blood and of the hematopoietic organs |
| 6 | HIV positive and full-blown AIDS |
| 7 | Non-active transplant recipients |
| 8 | Type 1 diabetes mellitus, complicated |
| 9 | Respiratory insufficiency / oxygen therapy |
| 10 | Neoplasia, active |
| 11 | Neuromyelitis optica |
| 12 | Immune haemolytic anaemias |
| 13 | Multiple sclerosis |
| 14 | Arterial vasculopathy |
| 15 | Certain conditions originating in the perinatal period |
| 16 | Type 2 diabetes mellitus, complicated |
| 17 | Chronic kidney failure |
| 18 | Endocrine, nutritional and metabolic diseases, and immunity disorders |
| 19 | Liver cirrhosis |
| 20 | Heart failure |
| 21 | Cushing's syndrome |
| 22 | Systemic sclerosis |
| 23 | Cerebral vasculopathy |
| 24 | Ankylosing spondylitis |
| 25 | Valvular heart disease |
| 26 | Chronic pancreatitis |
| 27 | Venous vasculopathy |
| 28 | Dementias |
| 29 | Ischaemic cardiopathy |
| 30 | Arrhythmic myocardiopathy |
| 31 | Diabetes insipidus |
| 32 | Cardiomyopathy (not arrhythmia-induced) |
| 33 | Parkinson's disease |
| 34 | Epilepsy |
| 35 | Pituitary dwarfism |
| 36 | Diseases of the circulatory system |
| 37 | Myasthenia gravis |
| 38 | Addison's disease |
| 39 | Chronic obstructive pulmonary disease |
| 40 | Rheumatoid arthritis |
| 41 | Psoriasis and psoriatic arthropathy |
| 42 | Diseases of the nervous system and sense organs |
| 43 | Chronic hepatitis |
| 44 | Type 1 diabetes mellitus |
| 45 | Systemic lupus erythematosus |
| 46 | Crohn's disease and ulcerative colitis |
| 47 | Alzheimer's disease |
| 48 | Hypercholesterolemia |
| 49 | Disease of the musculoskeletal system and connective tissue |
| 50 | Type 2 diabetes mellitus |
| 51 | Infectious and parasitic diseases |
| 52 | Sjögren's disease |
| 53 | Hyperparathyroidism and hypoparathyroidism |
| 54 | Diseases of the genitourinary system |
| 55 | Congenital anomalies |
| 56 | Hypertension |
| 57 | Neoplasia, follow-up |
| 58 | Diseases of the skin and subcutaneous tissue |
| 59 | Neoplasia, remission |
| 60 | Hypothyroidism |
| 61 | Rare cancer |
| 62 | Basedow's disease |
| 63 | Asthma |
| 64 | Hashimoto's disease |
| 65 | Diseases of the digestive system |
| 66 | Schizophrenia |
| 67 | Bipolar disorder |
| 68 | Depression |
| 69 | Personality disorder |

**Codes used for identifying the list of the 69 diseases and conditions candidate to be tested as predictors of five-year mortality.**

| Disease/condition | **ICD-10 code** | **ICD-9 CM code** | **DRG code** | **ATC code** | **Outpatient services code** | **Exemption code** |
| --- | --- | --- | --- | --- | --- | --- |
| Active transplant recipients |  |  | 302, 103, 495, 480, 481, 512, 513 |  |  | 052 |
| Dialysis |  |  |  |  | 39.95, 54.98 (at least 70 records) |  |
| Symptoms, signs and morbid conditions unspecified |  |  |  |  |  | RQ |
| Acromegaly and gigantism |  | 253.0 |  | H01CB (DDD>50%) |  | 001 |
| Diseases of the blood and of the hematopoietic organs |  |  |  |  |  | RD |
| HIV positive and full-blown AIDS |  | 042, V08 | 488, 489, 490 | J05AB14, J05AE, J05AG, J05AR, J05AX07, J05AX23, J05AX29, J05AJ01, J05AJ02, J05AJ03, J05AJ04 (DDD>30%) |  | 020 |
| Non-active transplant recipients |  | V42.0, V42.1, V42.6, V42.7, V42.8, V42.3, V42.4, V42.5, V42.9 | 302, 103, 495, 480, 481, 512, 513 |  |  | 052 |
| Type 1 diabetes mellitus, complicated |  | 250.41, 250.43, 250.51, 250.53, 250.61, 250.63, 250.71, 250.73, 250.81, 250.83, 250.91, 250.93, 362.01-363.07, 357.2 | 285 | N03AX16 or N03AX12 (DDD>30%) | 14.33, 14.34, 14.75, 965.91, 965.92, 965.93, 965.94, 965.95, 965.96 |  |
| Respiratory insufficiency / oxygen therapy |  |  | 518.83, 518.84 | V03AN01 (at least 400 €) |  | 024.518.81, 024.518.83 |
| Neoplasia, active |  | 140–208, V58.0, V58.1 99.25 (procedure) |  | L01 | 38.99.1, 38.99.2, 89.01.M, 89.7C.1, 92.24, 92.25.1, 92.27.1, 92.27.3, 92.27.5, 92.28.3, 92.28.4, 92.28.5, 92.28.6, 92.29.H, 92.29.J, 92.29.K, 92.29.L, 92.29.M, 99.25, MAC01, MAC02, MAC03, MAC04 | 046.340, 047.710.1, 048, RM0120 |
| Neuromyelitis optica |  | 341.0 |  |  |  | 041.341.0 |
| Immune haemolytic anaemias |  | 283.0 |  |  |  | 003 |
| Multiple sclerosis |  | 340, 341.9 | 013 | L03AB07, L03AB08, L03AX13, L04AA27, L04AA23, L04AA36, L04AA42, L04AA34, L04AA31, L04AA40, L04AX07 |  | 046.340 |
| Arterial vasculopathy |  | 440, 441, 442, 443.1, 443.2, 444, 445, 447 39.24, 39.25, 39.26, 39.50, 39.51, 39.52, 39.54, 39.56, 39.57, 39.58, 39.71, 39.72, 39.73, 39.74, 39.79, 39.90 (procedure) |  |  |  | 002.440, 002.441.2, 002.441.4, 002.441.7, 002.441.9, 002.442, 002.444, 002.447.0, 002.447.1, 002.447.6 |
| Certain conditions originating in the perinatal period |  |  |  |  |  | RP |
| Type 2 diabetes mellitus, complicated |  | 250.40, 250.42, 250.50, 250.52, 250.60, 250.62, 250.70, 250.72, 250.80, 250.82, 250.90, 250.92, 357.2, 362.01-363.07 | 285 | N03AX12, N03AX16 (DDD>30%) | 14.33, 14.34, 14.75, 96.59.1–96.59.6 |  |
| Chronic kidney failure |  | V56, 585, 586 |  | H05BX01, H05BX02, V03AE01, V03AE02, V03AE03 (DDD>50%) |  | 023, 031.403, 031.404, 0031.403, 0031.404, 061, 062 |
| Endocrine, nutritional and metabolic diseases, and immunity disorders |  |  |  |  |  | RC |
| Liver cirrhosis |  | 456.0, 456.1, 456.2, 571.2, 571.5, 571.6, 571.8, 572.3 | 316, 317 |  |  | 008 |
| Heart failure |  | 428 |  | C09A, C09C (DDD>50%) and [C03CA, C03CB, C03EB (DDD>50%) or C07AG02 C07AB02 C07AB07 (DDD>50%)] |  | 021.428 |
| Cushing's syndrome |  | 255.0 |  |  |  | 032 |
| Systemic sclerosis |  | 710.1 |  |  |  | 047, RM0120 |
| Cerebral vasculopathy |  | 430-438 |  |  |  | 002.433, 002.434, 002.437 |
| Ankylosing spondylitis |  | 720.0 |  |  |  | 054 |
| Valvular cardiopathy |  | 394-397, 745-747, V42.2, V43.3 35 (procedure) |  |  |  | 002.394, 002.395, 002.396, 002.397, 002.424, 002.745, 002.746, 002.747, 002.V42.2, 002.V43.3 |
| Chronic pancreatitis |  | 577.1 |  | A09AA (DDD>50%) |  | 042 |
| Venous vasculopathy |  | 452, 453, 459.1 |  |  |  | 002.452, 002.453, 002.459.1 |
| Dementias |  | 290, 294 |  |  |  | 011.290.0, 011.290.1, 011.290.2, 011.290.4, 011.291.1, 011.294.0 |
| Ischaemic cardiopathy |  | 410-414 36 (procedure) |  | C01DA (DDD>50%) |  | 002.414 |
| Arrhythmic myocardiopathy |  | 426, 427, V45.0 37.65, 37.66, 37.68, 37.70, 37.87, 37.89, 37.96 (procedure) |  | C01B (DDD>50%) | 89.48.1 | 002.426, 002.427, 002.V45.0 |
| Diabetes insipidus |  | 253.5 |  |  |  | 012 |
| Cardiomyopathy (not arrhythmia-induced) |  | 402, 404, 415-417, 425, 429.4 |  | C02KX01, C02KX02, C02KX03, G04BE03, G04BE08, B01AC09, B01AC27 (DDD>50%) |  | 031.402, 031.403, 002.416, 002.417, 002.429.4, 0031.402, 0031.403 |
| Parkinson's disease |  | 332.0, 332.1 |  | N04 (DDD>30%) |  | 038 |
| Epilepsy |  | 345 |  | N03AB02, N03AX14, N03AX18, N03AX23 (DDD>50%) |  | 017.345 |
| Pituitary dwarfism |  | 253.3 |  |  |  | 039 |
| Diseases of the circulatory system |  |  |  |  |  | RG |
| Myasthenia gravis |  | 358.0 |  | N07AA02 (DDD>50%) |  | 034.358.0, RFG101 |
| Addison's disease |  | 255.4 |  |  |  | 022 |
| Chronic obstructive pulmonary disease |  | 491, 492, 494, 496 |  | R03 (DDD>30%, Age ≥45) |  | 057 |
| Rheumatoid arthritis |  | 714.0, 714.1, 714.2, 714.30, 714.32, 714.33 |  |  |  | 006 |
| Psoriasis and psoriatic arthropathy |  | 696 |  |  |  | 045 |
| Diseases of the nervous system and sense organs |  |  |  |  |  | RF (except RFG101), 0031.362.11 |
| Chronic hepatitis |  | 70 |  | L03AB04, L03AB05, L03AB06, L03AB09, L03AB10, L03AB11, L03AB12, L03AB60, L03AB61 (DDD>50%) L04AX28 J05AE14, J0e5AX14, J05AX15, J05AX16, J05AX65, J05AX67, J05AX68 |  | 016 |
| Type 1 diabetes mellitus |  | 250.01, 250.03, 250.11, 250.13, 250.21, 250.23, 250.31, 250.33 | 295 | A10A (DDD>50%) |  | 013.250 (Age<35) |
| Systemic lupus erythematosus |  | 710.0 |  |  |  | 028 |
| Crohn's disease and ulcerative colitis |  | 555, 556 |  | A07EA (at least 2 records), L04AX08 |  | 009 |
| Alzheimer's disease |  | 331.0 |  | N06D (DDD>30%) |  | 029.331.0 |
| Hypercholesterolemia |  | 272.0, 272.2, 272.4, 272.9 |  | C10AA (DDD>50%) |  | 025 |
| Disease of the musculoskeletal system and connective tissue |  |  |  |  |  | RM (except RM0120), 060, 067 |
| Type 2 diabetes mellitus |  | 250.00, 250.02, 250.10, 250.12, 250.20, 250.22, 250.30, 250.32 | 294 | A10B (DDD>50%) |  | 013.250 (Age ≥35) |
| Infectious and parasitic diseases |  |  |  |  |  | RA |
| Sjogren’s disease |  | 710.2 |  |  |  | 030 |
| Hyperparathyroidism and hypoparathyroidism |  | 252.0, 252.1 |  |  |  | 026 |
| Diseases of the genitourinary system |  |  |  |  |  | RJ, 063 |
| Congenital anomalies |  |  |  |  |  | RN, 066, 065, 064 |
| Hypertension |  | 401, 403, 405 | 134 | C02AC01, C02CA04, C03, C07, C08C, C09 (DDD>50%) |  | 031.401, 031.405, D31.401, D31.405, 0031, 0031.405.0 |
| Neoplasia, follow-up |  | 140-208, V58.0, V58.1, 99.25 (procedure) |  | L01, L02 | 38.99.1, 38.99.2, 89.01.M, 89.7C.1, 92.24, 92.25.1, 92.27.1, 92.27.3, 92.27.5, 92.28.3, 92.28.4, 92.28.5, 92.28.6, 92.29.H, 92.29.J, 92.29.K, 92.29.L, 92.29.M, 99.25, MAC01, MAC02, MAC03, MAC04 | 046.340, 047.710.1, 048, RM0120 |
| Diseases of the skin and subcutaneous tissue |  |  |  |  |  | RL (except RL0020) |
| Neoplasia, remission |  | 140-208, V58.0, V58.1, 99.25 (procedure) |  | L01 (except L01AB01, L01AA01), L01AB01, L01AA01 (5≤Age≤10) | 99.25, 92.24, 92.25.1, 92.27.1, 92.27.3, 92.27.5, 92.28.3, 92.28.4, 92.28.5, 92.28.6, 38.99.1, 38.99.2, 89.01.M, 89.7C.1, 92.29.H, 92.29.J, 92.29.K, 92.29.L, 92.29.M, MAC01, MAC02, MAC03, MAC04 | 048, 047.710.1, 046.340, RM0120 |
| Hypothyroidism |  | 243, 244 |  | H03AA01 (DDD>30%) |  | 027 |
| Rare cancer |  |  |  |  |  | RB |
| Basedow's disease |  | 242.0, 242.1, 242.2, 242.3 |  |  |  | 035 |
| Asthma |  | 493 |  | R03DC03, R03DC01 (DDD>40%), R03 (DDD>30%) Age<45 |  | 007.493 |
| Hashimoto's disease |  | 245.2 |  |  |  | 056 |
| Diseases of the digestive system |  |  |  |  |  | RI, 059 |
| Schizophrenic disorder | F20, F25, F28, F29 | 295, 297,  298.2, 298.3, 298.8,  298.9, 298.4 in  psychiatric ward |  | N05AB02,  N05AD0,  N05AF01,  N05AF05,  N05AG01,  N05AH03,  N05AH03,  N05AX08,  N05AX13,  N05AX12,  N05AH02  (at least 3 months)  + **exclusion A** | 295, 297, 298.2, 298.3, 298.8, 298.9, 298.4 in  psychiatric ward | 044.295, 044.297,  044.298.2, 044.298.3,  044.298.8, 044.298.9,  044.298.4 |
| Bipolar disorder | F30, F31, F34.0, F38.0 | 296, 296.1,  296.4, 296.5, 296.6,  296.7, 296.8, 296.81,  296.89, 296.99, 298.1 |  | N05AN  (at least one record)  + **exclusion B**  Or  N03AF01,  N03AG01,  N03AX09 (at least 3 months)  Or  less than 3 months if  N05AN,  N03AF01,  N03AG01,  N03AX09  more than 3 months in the last five years + **exclusion C**  Or  N05A at least 3 months  and N06A,  N05AN,  N03AF01,  N03AG01,  N03AX09  + **exclusion D** | 296, 296.1, 296.4, 296.5, 296.6, 296.7, 296.8, 296.81, 296.89,  296.99, 298.1 | 044.296, 044.296.1,  044.296.4, 044.296.5,  044.296.6,  044.296.7, 044.296.8,  044.296.81,  044.296.89,  044.296.99, 044.298.1 |
| Depressive disorder | F32, F33, F34.1, F34.8, F34.9, F38.1,  F38.8, F39, F43.1, F43.2, | 296.2, 296.3,  296.82, 296.9, 298,  300.4, 309, 309.1,  311.* |  | N06A more than 2 weeks  Or  N06A less than 2 weeks if more than 2 weeks in the last 5 years    + **exclusion E** | 296.2, 296.3, 296.82, 296.9,  298, 300.4, 309,  309.1, 311 | 044.296.2, 044.296.3,  044.296.82, 044.296.9, 044.298, 044.300.4, 044.309,  044.309.1, 044.311 |
| Personality disorder | F60, F61 | 301 in  in  Psychiatric ward |  |  | 301 | 301 |

# ICD-10: International Classification of Diseases, Tenth Revision; ICD-9 CM: International Classification of Diseases, Ninth Revision, Clinical Modification; DRG: Diagnosis-Related Group; ATC: Anatomical Therapeutic Chemical classification system; DDD: Defined Daily Dose; EX: exemption code

A NHS beneficiary is classified as suffering from a given condition according to whether in the period before the index year at least one of these events occurred at least once:

- inpatients primary diagnosis, or co-existing condition, recorded according to at least one of the corresponding ICD-10, ICD-9 CM and/or DRG codes
- outpatient drug prescription, recorded according to at least one of the corresponding ATC codes; with the aim of avoiding that a sporadic drug dispensation lead to classify a patient affected by the considered condition (false positive diagnoses) a restraint was introduced, that is the proportion of days covered (ratio between the total amount of the Defined Daily Dose dispensed packing in the previous two years and 730 days) has to be greater than 30% (e.g., Parkinson's disease) or 50% (e.g., heart failure) for classifying the beneficiary affected from the condition;
- outpatient services, recorded according to the corresponding regional nomenclator;
- exemption to co-payment for chronic disease, recorded according to the corresponding national exceptions coding

**Notes:**

- **Exclusion A:**

1) Presence of a different diagnosis in Mental Health Information System (ICD10 codes F*), SDO-DRG (ICD9-CM codes 290-319) and Exemption code (290-319)

Or

2) Presence of an antidepressant drug (ATC codes N06A) or Valproate (ATC codes N03AG01), Carbamazepine (ATC codes N03AF01), Lamotrigine (ATC codes N03AX09) in the last 5 years (from 01/01/2010 to 01/01/2015)

- **Exclusion B:**

1) Presence of a different diagnosis in Mental Health Information System (ICD10 codes F*), SDO-DRG (ICD9-CM codes 290-319) and Exemption code (290-319)

- **Exclusion C:**

1) Presence of epilepsy (ICD-9 CM codes 345.*)

2) Presence of a different diagnosis in Mental Health Information System (ICD10 F*), SDO-DRG (290-319) and Exemption code (290-319)

- **Exclusion D:**

1) No treatment for more than 3 months with an antipsychotic drug in the previous five years (ATC codes N05A) or Valproate (ATC codes N03AG01), Carbamazepine (ATC codes N03AF01), Lamotrigine (ATC codes N03AX09)

2) Presence of a different diagnosis in Mental Health Information System (ICD10 F*), SDO-DRG (290-319) and Exemption code (290-319)

- **Exclusion E:**

1) Presence of a different diagnosis in PSICHEWEB, SDO-DRG and Exemption code

2) Presence of treatment in the previous five years for more than 3 months with an antipsychotic drug (ATC codes N05A) or Valproate (ATC codes N03AG01), Carbamazepine (ATC codes N03AF01), Lamotrigine (ATC codes N03AX09)

- Diabetes. Type 1 (T1DM) and type 2 (T2DM) diabetes mellitus are mutually exclusive categories. The following rules established the condition for each patient.
  - if T2DM_ATC = 1 then T2DM
  - if T2DM_ATC = 0 and T1DM_ATC = 0 and (T1DM_DRG = 1 or T1DM_ICD9 = 1 or T1DM_EX = 1 or T2DM_DRG = 1 or T2DM_ICD9 = 1 or T2DM_EX = 1) then T2DM
  - if (T1DM_EX = 1 or T1DM_DRG = 1 or T1DM_ICD9 = 1) and T1DM_ATC = 1 then T1DM
  - if T1DM_ATC = 1 and T2DM_EX = 1 then T2DM
  - if (T2DM_DRG = 1 or T2DM_ICD9 = 1) and T1DM_ATC = 1 then T2DM
  - if T1DM_ATC = 1 then T1DM
- Neoplasia (active, follow-up and remission). ATC codes L01AB01 and L01AA01 should not be considered if there is an exemption with the following codes: 006.710, 045.696, 028.710, 047.710.1, 046.340
- Respiratory insufficiency / oxygen therapy, Chronic obstructive pulmonary disease, Asthma are mutually exclusive categories. The following rules established the condition for each patient.
  - if ASTHMA_EX = 1 then ASTHMA
  - if ASTHMA_ATC1 = 1 then ASTHMA
  - if ASTHMA_ICD9 = 1 then ASTHMA
  - if ASTHMA_ATC2 = 1 then ASTHMA
  - if COPD_ATC = 1 then COPD
  - if COPD_ICD9 = 1 then COPD
  - else if Respiratory insufficiency / oxygen therapy.
- According to the following table, the right condition (condition 2) was set to “absent” if patient suffered from the left one (condition 1)

| **Condition 1** | **Condition 2** |
| --- | --- |
| Arrhythmic myocardiopathy | Hypercholesterolemia |
| Arrhythmic myocardiopathy | Hypertension |
| Type 2 diabetes mellitus, complicated | Type 2 diabetes mellitus |
| Type 2 diabetes mellitus, complicated | Hypercholesterolemia |
| Type 1 diabetes mellitus, complicated | Type 1 diabetes mellitus |
| Type 1 diabetes mellitus, complicated | Hypercholesterolemia |
| Cardiomyopathy (not arrhythmia-induced) | Hypercholesterolemia |
| Cardiomyopathy (not arrhythmia-induced) | Hypertension |
| Cerebral vasculopathy | Hypertension |
| Cerebral vasculopathy | Hypercholesterolemia |
| Dementias | Cerebral vasculopathy |
| Dementias | Parkinson's disease |
| Dementias | Epilepsy |
| Dementias | Alzheimer's disease |
| Dementias | Hypertension |
| Dementias | Hypercholesterolemia |
| Dialysis | Chronic kidney failure |
| Dialysis | Hypertension |
| Type 1 diabetes mellitus | Hypercholesterolemia |
| Ankylosing spondylitis | Psoriasis and psoriatic arthropathy |
| Respiratory insufficiency / oxygen therapy | Chronic obstructive pulmonary disease |
| Respiratory insufficiency / oxygen therapy | Asthma |
| Heart failure | Ischaemic cardiopathy |
| Heart failure | Valvular heart disease |
| Heart failure | Arrhythmic miocardiopathy |
| Heart failure | Cardiomyopathy (not arrhythmia-induced) |
| Heart failure | Hypercholesterolemia |
| Heart failure | Hypertension |
| Ischaemic cardiopathy | Hypercholesterolemia |
| Ischaemic cardiopathy | Hypertension |
| Chronic kidney failure | Hypertension |
| Systemic sclerosis | Disease of the musculoskeletal system and connective tissue |
| Valvular heart disease | Hypercholesterolemia |
| Valvular heart disease | Hypertension |
| Rheumatoid arthritis | Psoriasis and psoriatic arthropathy |
| Rheumatoid arthritis | Sjögren's disease |
| Neoplasia, active | Neoplasia, follow-up |
| Neoplasia, active | Neoplasia, remission |
| Arterial vasculopathy | Hypertension |
| Arterial vasculopathy | Hypercholesterolemia |
| Neoplasia, follow-up | Neoplasia, remission |
| Liver cirrhosis | Chronic hepatitis |
| Systemic lupus erythematosus | Disease of the musculoskeletal system and connective tissue |
| Chronic obstructive pulmonary disease | Asthma |
| Active transplant recipients | Non-active transplant recipients |
| Hypothyroidism | Basedow's disease |
| Hypothyroidism | Hashimoto's disease |
| Alzheimer's disease | Cerebral vasculopathy |
| Alzheimer's disease | Parkinson's disease |
| Alzheimer's disease | Epilepsy |
| Alzheimer's disease | Hypercholesterolemia |
| Alzheimer's disease | Hypertension |
| Sjögren's disease | Disease of the musculoskeletal system and connective tissue |
| Type 2 diabetes mellitus | Hypercholesterolemia |
| Schizophrenia | Bipolar disorder |
| Schizophrenia | Personality disorder |
| Schizophrenia | Depression |
| Bipolar disorder | Personality disorder |
| Bipolar disorder | Depression |
| Personality disorder | Depression |

**Figure S1. Flow-chart of cohort selection.**

**10,091,974**

Beneficiaries of the Regional Health Service (RHS) on 01/01/2015

**8,126,963**

Included in the study cohort

Excluded:

**101,157 not resident in Lombardy Region**

**197,679 beneficiaries of the RHS for less than five years**

**1,606,765 aged less than 18 years**

**59,680 institutionalized**

**Figure S2. Comparison between the ROC curves of the original CReSC and the update version (CReSc-2.0).**
